# Infective Endocarditis in Northeastern Thailand

**DOI:** 10.3201/eid2003.131059

**Published:** 2014-03

**Authors:** George Watt, Orathai Pachirat, Henry C. Baggett, Susan A. Maloney, Viraphong Lulitanond, Didier Raoult, Saithip Bhengsri, Somsak Thamthitiwat, Anucha Paupairoj, Michael Kosoy, Nongrak Ud-Ai, Wichuda Sukwicha, Toni Whistler, Pierre-Edouard Fournier

**Affiliations:** Thailand Ministry of Public Health–US Centers for Disease Control and Prevention Collaboration, Nonthaburi, Thailand (G. Watt, H.C. Baggett, S.A. Maloney, S. Bhengsri, S. Thamthitiwat, W. Sukwicha, T. Whistler);; Khon Kaen University Faculty of Medicine, Khon Kaen, Thailand (O. Pachirat, V. Lulitanond, A. Paupairoj, N. Ud-Ai);; Aix Marseille Université, Marseille, France (D. Raoult, P.-E. Fournier);; Centers for Disease Control and Prevention, Atlanta, Georgia, USA (H.C. Baggett, S.A. Maloney, T. Whistler);; Centers for Disease Control and Prevention, Fort Collins, Colorado, USA (M. Kosoy)

**Keywords:** Infective endocarditis, congestive heart failure, Thailand, zoonoses, Bartonella, Q fever, Viridans streptococci, Coxiella burnetii, bacteria, antimicrobial drugs, rheumatic heart disease, heart valve

## Abstract

Despite rigorous diagnostic testing, the cause of infective endocarditis was identified for just 60 (45.5%) of 132 patients admitted to hospitals in Khon Kaen, Thailand, during January 2010–July 2012. Most pathogens identified were *Viridans streptococci* and zoonotic bacteria species, as found in other resource-limited countries where underlying rheumatic heart disease is common.

Serologic testing of patients with blood culture–negative endocarditis has identified *Coxiella burnetii,* the causative agent of Q fever, and *Bartonella* spp. as noteworthy causes of infective endocarditits (IE) in resource–limited countries (*1*–*4*). Many cases of IE that were not diagnosed by standard blood culture were caused by zoonotic bacteria (*5*). Prospective, systematic descriptions of the etiology and characteristics of IE in Southeast Asia are lacking. We therefore collected detailed clinical, laboratory, and epidemiologic information for patients with confirmed IE in Khon Kaen, Thailand, and conducted specialized testing methods in addition to standard blood cultures to facilitate assessment for zoonotic and nonzoonotic bacteria as the cause of IE.

## The Study

 During January 25, 2010–July 19, 2012, patients were prospectively enrolled in this study at 2 tertiary care referral hospitals located on the campus of the medical school of Khon Kaen University. Srinagarind Hospital is a 777-bed general hospital, and the Queen Sirikit Heart Center of the Northeast is a 200-bed specialized cardiac center in which ≈10 heart valve replacement surgeries are performed each month. Patients with suspected IE are referred from much of northeastern Thailand, a region of ≈21 million persons.

Transthoracic echocardiography was performed for patients suspected of having IE; consenting patients >16 years of age who met modified Duke criteria for endocarditis were enrolled in this study. Underlying cardiac conditions were assessed by cardiologists on the basis of patients’ medical records, history, physical examination, and echocardiographic findings. At admission to a hospital, 3 separate blood samples for culture were obtained in <90 minutes. Blood was inoculated into aerobic medium (BD BACTEC Plus Aerobic/F Medium; Becton Dickinson, Franklin Lakes, NJ, USA), and cultures were processed by using an automated system (BD Bactec Fx series; Becton Dickinson). Pathogens were identified to species level whenever possible, but some blood culture isolates were defined only to the genus level (e.g., viridans group streptococci). One month after admission, a convalescent-phase serum specimen was obtained from each study patient and these patients were evaluated by a cardiologist.

Acute- and convalescent-phase serum specimens were tested for *C. burnetii* and *Legionella pneumophila* by indirect immunofluorescence assay (IFA) as described (*5*). Phase 1 IgG reciprocal titers >800 for *C. burnetii* and total antibody reciprocal titers ≥256 for *L. pneumophila* on either serum specimen were defined as positive (*5*). IFA IgG reciprocal titers of ≥800 to *Bartonella quintana* and *Bartonella henselae* were deemed positive. Specific antibodies to *Brucella melitensis* and *Mycoplasma pneumoniae* were detected with a commercial immunoenzymatic antibody test (*Brucella* antibody and Platellia *M. pneumoniae* IgM kits, respectively; Bio-Rad, Marnes-la-Coquette, France). Reciprocal titers ≥200 were considered positive. DNA was extracted from surgically excised heart valves by using the QIAamp DNA FFPE Tissue Kit (QIAGEN, Courtaboeuf, France) as described by the manufacturer. Previously described broad spectrum PCR primers and amplification and sequencing conditions (*4*) were used to detect all bacteria (16S rRNA); all fungi (18S rRNA); *Staphylococcus aureus*, mitis and gallolyticus group streptococci, *Enterococcus faecalis* and *E. faecium*, *Mycoplasma hominis*, *C. burnetii*, *Bartonella* spp., and *Tropheryma whipplei*.

Table 1 describes characteristics of the 132 enrolled patients: the median age was 47 years (range 16–85) and 68.9% were male. Most of the study patients lived in rural areas, most had a history of animal contact, and most were farmers of rice or vegetables. All study patients had definite IE as determined by using modified Duke criteria. Patients had high fever and were severely ill at admission; more than half had congestive heart failure (Table 1). Underlying cardiac pathologic changes were identified in 96 (72.7%) study patients (Table 1); rheumatic heart disease (RHD) was the most common condition, as has been found in other resource-limited countries (*1*,*2*,*6*,*7*). RHD was identified in 37 (28.0%) of the 132 patients overall; the 37 represented 38.5% of the 96 patients with identified underlying cardiac pathologic changes. IE was detected on prosthetic heart valves in 12 (9.9%) of the study patients, including 2 patients whose blood cultures grew coagulase-negative staphylococci.

**Table 1:** Characteristics of 132 patients with infective endocarditis, Khon Kaen, Thailand, January 2010–July 2012

Characteristic	No. patients (%)*
Demographic	
Median age, y (range)	47 (16–85)
Male sex	91 (68.9)
Live in rural area	111 (84.1)
Occupation	
Poultry farmer	74 (56.1)
Livestock farmer	39 (29.6)
Rice farmer	59 (44.7)
Vegetable farmer	28 (21.2)
Housewife	9 (6.8)
Animal contact	
Own pets	92 (69.7)
Own livestock	46 (34.9)
Own poultry	89 (67.4)
Underlying cardiac pathologic changes	
Identified underlying cardiac condition	96 (72.7)
Rheumatic heart disease	37 (28.0)
Mitral valve prolapse	19 (14.4)
Prosthetic heart valve	13 (9.9)
Congenital heart disease	11 (8.3)
Degenerative valve disease	9 (6.8)
Other cardiac conditions	4 (3.0)
Clinical findings	
Type of heart valves involved	
Native	120 (91.1)
Prosthetic	12 (9.9)
Heart valves involved overall, n = 132	
Aortic	62 (47.0)
Mitral	52 (39.4)
Tricuspid	2 (0.2)
Aortic and mitral	10 (7.6)
Mitral and pulmonary	1 (0.01)
Aortic and tricuspid	1 (0.01)
Mitral and tricuspid	1 (0.01)
Aortic, mitral, and tricuspid	1 (0.01)
Heart valves involved in patients with underlying rheumatic heart disease, n = 37	
Aortic	21 (56.8)
Mitral	10 (27.0)
Mitral and aortic	5 (13.5)
Mitral, aortic, and tricuspid	1 (2.7)
Days of fever before admission, median (range)	21 (0–270)
Patients receiving antibacterial drugs during week before admission	77 (58.3)
Mean ± SD maximum temperature during first 24 h after admission	38.3°C.± 0.9°C
Congestive heart failure at admission†	64 (48.5)

*Data indicate no. (%) patients with specified characteristic unless otherwise indicated. †Diagnosis was made by a cardiologist.

Of the 132 study patients, 100 (75.8%) underwent surgery. Such management reflects current international guidelines for complicated IE, which emphasize identifying high-risk patients, transferring them to a specialized medical–surgical center, and performing early valvular surgery (*8*). Within 1 month, 11 (8.3%) of the 132 patients died and 6 were still hospitalized; 5 died after discharge. Four additional patients are known to have died after leaving the study, but we did not systematically assess survival beyond the 1-month follow-up examination. The case-fatality proportion calculated on the basis of known deaths was 11.4%, but the possibility of additional fatalities cannot be excluded. The mortality rate was 18.8% for patients who did not undergo surgery and 9.0% for those who did.

A pathogen was identified for 60 (45.5%) cases (Table 2). The etiologic agent was identified for 7 (21.9%) of 32 nonsurgical cases, 5 by blood culture and 2 by IFA, compared with 53 (53.0%) of the 100 study patients who underwent surgery (Fisher exact test, p = 0.02). Among patients who underwent surgery, diagnosis was made by PCR of heart valve tissue for 29, by blood culture for 11, and by >1 method for the remaining 13; no discrepancies between results from different diagnostic modalities were found. Many (80.3%) patients had been referred from other hospitals for management of IE and were already being treated for that illness. We confirmed that 77 (58.3%) patients had received antibacterial drugs during the week before admission. We suppose that the low diagnostic yield was associated with antibacterial drug use, but more detailed information is needed to determine the accuracy of this supposition.

**Table 2:** Bacterial species identified in 60 patients with infective endocarditis, Khon Kaen, Thailand, January 2010–July 2012*

Disease type, organisms	No. (%) cases
Zoonoses	15 (25)
* Coxiella burnetti*†	5
* Bartonella henselae*†	4
* Streptococcus suis*	4
* Erysipelothrix rhusiopathiae*†	1
* Campylobacter fetus*†	1
Nonzoonoses	45 (75)
* Enterococcus faecalis*	6
* Enterococcus *spp.	4
* Staphylococcus aureus*	5
Coagulase–negative* Staphylococcus *sp.	2
Viridans streptococci	5
* Streptococcus agalactiae*	4
* Streptococcus anginosus*	4
* Streptococcus gordonii*	2
* Streptococcus gallolyticus*†	2
* Streptococcus mitis*	2
* Streptococcus dysgalactiae*	1
* Streptococcus oralis*	1
* Streptococcus difficilis*†	1
* Streptococcus pneumonia*	1
* Streptococcus sinensis*†	1
* Streptococcus *spp.	1
* Corynebacterium diptheriae*	1

*No pathogenic agents were identified for 72 (54.6%) of 132 patients. †Not previously reported to cause infective endocarditis in Thailand.

Among the 60 cases of IE for which a causative pathogen was identified, 15 (25.0%) were attributed to zoonotic bacteria (Table 2): *C. burnetti* (5 cases), *B. henselae* (4 cases), *Streptococcus suis* (4 cases), *Erysipelothrix rhusiopathiae* (1 case), and *Campylobacter fetus* (1 case). *B. quintana* causes ≈75% of *Bartonella* IE cases worldwide (*9*) but was not found in this study. Verification that *C. burnetti* (*10*), *Bartonella spp.* (*11*), *E. rhusiopathiae*, and *C. fetus* cause IE in Thailand highlight the noteworthiness of emerging zoonotic pathogens in this region. The identification of Q fever IE stimulated efforts by public health authorities of Thailand to characterize Q fever prevalence and transmission nationwide.

Streptococci generally cause the majority of infections of native heart valves (*12*), and 90.1% of patients in this study had native valve involvement. Viridans streptococci, including *S. suis*, accounted for 43.3% of cases of known etiology (Table 2) and included 3 species of streptococci not previously reported to cause IE in Thailand (*S. gallolyticus*, *S. difficilis*, and *S. sinensis*). In Thailand, as elsewhere, health care–associated IE and IE among intravenous drug users is often caused by *S. aureus* (*13*,*14*). None of the patients in this study were known to be intravenous drug users, and few had health care–associated infections; 5 (8.3%) of 60 cases were caused by *S. aureus*.

## Conclusions

Zoonotic bacteria were detected by specialized testing of 11.4% of IE patients in northeastern Thailand, but an etiology could not be determined for more than half (54.5%) of the patients. Infections with viridans streptococci predominated among cases of known etiology, and RHD was the most common underlying pathologic change. To increase diagnostic yield, we recommend intensification of efforts to obtain blood cultures in the absence of antimicrobial drugs and increase of laboratory capacity to test for zoonotic bacteria (*15*).
